# The Development, Validation, and Application of a UHPLC-HESI-MS Method for the Determination of 17 Cannabinoids in *Cannabis sativa* L. *var. sativa* Plant Material

**DOI:** 10.3390/molecules28248008

**Published:** 2023-12-08

**Authors:** Joanna Kanabus, Marcin Bryła, Marek Roszko

**Affiliations:** Department of Food Safety and Chemical Analysis, Prof. Waclaw Dabrowski Institute of Agricultural and Food Biotechnology—State Research Institute, Rakowiecka 36, 02-532 Warsaw, Poland

**Keywords:** *Cannabis sativa* L., cannabinoids, hemp tea, UHPLC-HESI-MS, validation

## Abstract

Cannabinoids are an important group of secondary metabolites found in the plant *Cannabis sativa* L. The growing interest in the use of hemp in food production (e.g., hemp teas, hemp cookies) makes it necessary to develop a method for determining these compounds in the plant, both fresh and dried. The selection of a suitable extraction liquid for the extraction of cannabinoids and the development of a method for the determination of 17 cannabinoids is a prelude to the development of an effective method for the extraction of these compounds. In the present study, a novel, simple, and efficient method was developed and validated for the determination of up to 17 cannabinoids in fresh plant parts (inflorescences and leaves) of *Cannabis sativa* L. and in dried material, including hemp teas. Analyses were performed using ultra-high-performance liquid chromatography-Q-Exactive Orbitrap mass spectrometry setup operating with a heated electrospray interface (UHPLC-HESI-MS). Based on the comparison, methanol was selected as the best for the extraction of cannabinoids from fresh and dried material. The efficiency and validity of the method were assessed using certified reference material (dried Cannabis) and confirmed by z-score from participation in an international proficiency test conducted by ASTM International for dried hemp.

## 1. Introduction

Hemp (*Cannabis sativa* L.) is a very versatile plant and one of the oldest crops in agriculture. The plant has been used to make paper and textiles, and its seeds are a source of fatty acids, amino acids, and fiber [1]. Cannabinoids are synthesized in the plant’s glandular trichomes, which are found in the female inflorescences. They are meroterpenoids with resorcinol cores containing an isoprenyl, alkyl, or aralkyl side chain in the para position. More than 120 cannabinoids are known; the best-known are Δ^9^-tetrahydrocannabinol (Δ^9^-THC) and cannabidiol (CBD). Δ^9^-THC has been classified as a controlled substance due to its confirmed psychoactive properties. CBD does not exhibit psychoactive properties; instead, it is believed to have analgesic and anti-inflammatory properties [2]. A key element in evaluating the safety of hemp products is assessing the presence of trace amounts of the psychoactive “total Δ^9^-THC”. The term “total Δ^9^-THC” refers to the sum of Δ^9^-tetrahydrocannabinol (Δ^9^-THC) and Δ^9^-tetrahydrocannabinolic acid (Δ^9^-THCA-A), which is readily converted to Δ^9^-THC by heat treatment [1]. The limits set by European Commission (EU) Regulation (EU) 2023/915 are for maximum levels of total Δ^9^-THC in seeds and products derived from them (hemp seed oil—7.5 mg kg^−1^ and seeds and products derived from seeds—3 mg kg^−1^) [3]. The European Food Safety Authority (EFSA) set an acute reference dose (ARfD) of 1 µg kg^−1^ body weight for “total Δ^9^-THC” [4]. The current changing regulations provide many facilitations and opportunities, due to which the market for hemp products is growing faster and faster.

The most important and first step in the determination of cannabinoids is their extraction. In recent years, many papers have been focused on technologies for extracting bioactive compounds from *Cannabis sativa* L., including cannabinoids. One of the conventional methods of extracting cannabinoids is liquid–solid extraction, such as maceration or percolation [5]. However, there is a problem with finding a standard for the method of extracting cannabinoids from fresh or dried *Cannabis sativa* L. There is much information regarding the extraction liquids used during cannabinoid extraction. On the other hand, there is a lack of appropriate comparison of the extraction liquids used to this day to identify the most suitable one for extracting cannabinoids from both fresh and dried plant material. According to several literature reports, the most suitable solvent for cannabinoid extraction is methanol (MeOH) or a mixture of MeOH and chloroform (CHCl_3_) in a ratio of 9:1 (*v*/*v*). However, chloroform is not recommended as a solvent due to its volatility and high toxicity [6,7,8]. According to Chen et al. [9], extraction of cannabinoids with ethanol (EtOH) is associated with lower extraction recoveries compared to a mixture of MeOH and CHCl_3_. The problem is related to the difficulty in separating the extracted components, as many other substances present in the plant (e.g., fats and waxes) are extracted with EtOH along with cannabinoids [9]. Compared to traditional solvent extraction, supercritical fluid extraction, such as carbon dioxide, allows for easier recovery of cannabinoids with low solvent (CO_2_) losses. The extract obtained by this method also requires further purification to remove lipids and waxes [9,10]. Attempts have been made to extract cannabinoids by other techniques. Baranauskaite et al. [11] compared maceration, ultrasound-assisted extraction (UAE), and heat reflux extraction (HRE). The authors concluded that UAE is the optimal extraction technique due to the low time, energy, and cost requirements. According to Brighenti et al. [12], dynamic maceration with EtOH was a more efficient method for extracting cannabinoids from *Cannabis sativa* L. compared to UAE and microwave-assisted extraction (MAE). The lack of specific regulations regarding the conduct of cannabinoid extraction and the methods used causes differences in the results obtained from extracting these compounds from similar matrices [13]. Taking into account the existing differences in the extraction liquids and methods used, this study undertook the development of an easy extraction method using different extraction liquids to compare the number of cannabinoids that can be extracted from plant material.

Gas chromatography coupled with mass spectrometry (GC-MS) is one of the most widely used techniques in the quantitative analysis of cannabinoids. However, using this technique, it is only possible to determine the total content of a given cannabinoid, which is the sum of the acidic and neutral components, because acidic cannabinoids undergo decarboxylation as a result of elevated temperatures during the separation step on the column. An additional complication is the need for compound derivatization [14,15]. The use of liquid chromatography coupled to mass spectrometry (LC-MS) does not require an initial derivatization process before analysis, and no decarboxylation of acidic compounds occurs during this process. This technique is a favorable alternative to GC. Using the LC-MS technique, which exhibits high sensitivity to cannabinoids, it is possible to achieve lower limits of quantification than with the GC-MS technique [1,2].

The aim of this study was (1) to develop a procedure for the extraction of compounds depending on the matrices studied, (2) to select appropriate chromatographic separation parameters for the analytes studied and analysis using an ultra-high-performance liquid chromatography-Q-Exactive Orbitrap mass spectrometry setup operating with a heated electrospray interface, (3) to validate the method, (4) to evaluate the suitability of the technique for assessing the content of cannabinoids in different parts of the *Cannabis sativa* L. *var. sativa* plant, and (5) to evaluate the content of 17 cannabinoids in commercially available products based on *Cannabis sativa* L. *var. sativa*. This is one of the first studies on the analysis of 17 cannabinoids in plant material.

## 2. Results

### 2.1. Optimization of the Procedure for the Extraction of Cannabinoids from the Tested Matrices

#### 2.1.1. Fresh Parts of Plants

**Figure 1:**
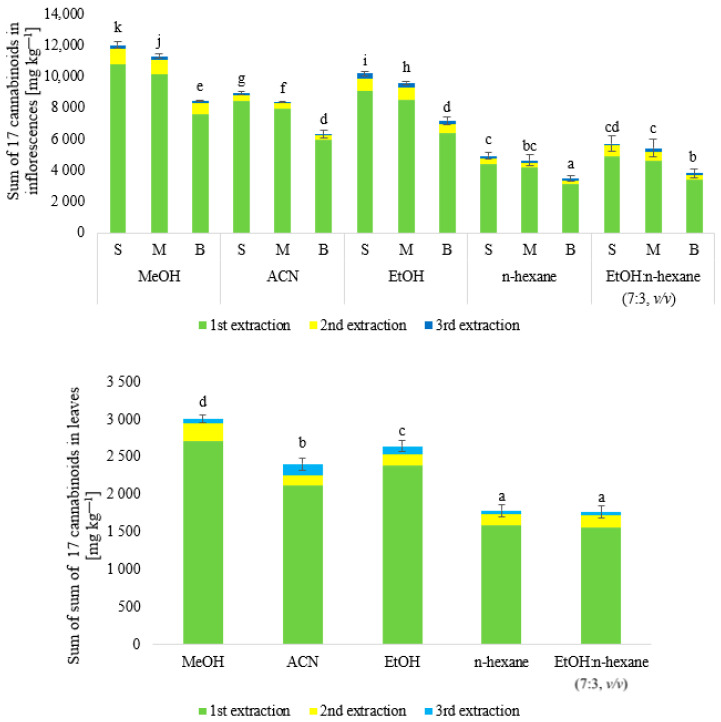
Comparison of extracted content of cannabinoids using different solvents from different parts of plant *Cannabis sativa* L. *var. sativa* (S—small inflorescences; M—medium inflorescences; B—big inflorescences) and leaves. a−k—symbol indicates a statistically significant result; *n* = 3.

The extraction efficiency of the sum of 17 cannabinoids from inflorescences (small, medium, and big) and leaves was compared using different extraction solvents (MeOH, ACN, EtOH, n-hexane, and EtOH:n-hexane mixture (7:3, *v*/*v*)). A three-step extraction was performed using each extraction solvent (5 mL), and the sum of 17 cannabinoids extracted at each extraction step was compared (Figure 1).

The sum of cannabinoids from three extractions with MeOH was taken as the possible maximum amount to be extracted from fresh material. When n-hexane and a mixture of EtOH:n-hexane (7:3, *v*/*v*) were used, the recoveries obtained were <70% and were significantly the lowest in terms of the extracted sum of cannabinoids. Based on the obtained results, these two types of extraction liquids were rejected. The first extraction of inflorescences resulted in the extraction of an average of 90% (MeOH), 70% (ACN), and 75% (EtOH) of all analyzed cannabinoids present in the inflorescences. Performing a second extraction allowed for the extraction of 8% (MeOH), 3% (ACN), and 6% (EtOH) more cannabinoids. The third extraction step yielded 2% (MeOH), 0.03% (ACN), and 0.06% (EtOH) cannabinoids. For the leaves, the use of MeOH extracted 92% of the cannabinoids in the first extraction step, 6% in the second, and 2% in the third step. The use of ACN or EtOH to extract 17 cannabinoids from the leaves in the first extraction stage allowed an average of 70 and 79% extraction efficiency, respectively. In the second stage of extraction, 5% of cannabinoids were extracted for two variants of extraction solvents, and in the third stage, 0.05 and 0.04% were extracted. Based on the results, it was concluded that the most suitable extraction liquid for the extraction of 17 cannabinoids from both inflorescences and leaves was MeOH, as the results showed statistically significant differences (*p* < 0.01) between the contents of the tested compounds in MeOH, ACN, and EtOH extracts, as well as the extraction efficiency of the given solvent (Figure 1). It was also found that two-stage extraction ensured the extraction of a satisfactory sum of cannabinoids from inflorescences and leaves. In addition, significant differences were observed in the sums of the cannabinoids in the different elements of the plant (inflorescences of different sizes and leaves) (Figure 1). In the case of MeOH extracts, the highest contents of the sum of these compounds were characterized by extracts of small and medium-sized inflorescences.

#### 2.1.2. Dried Plants

**Figure 2:**
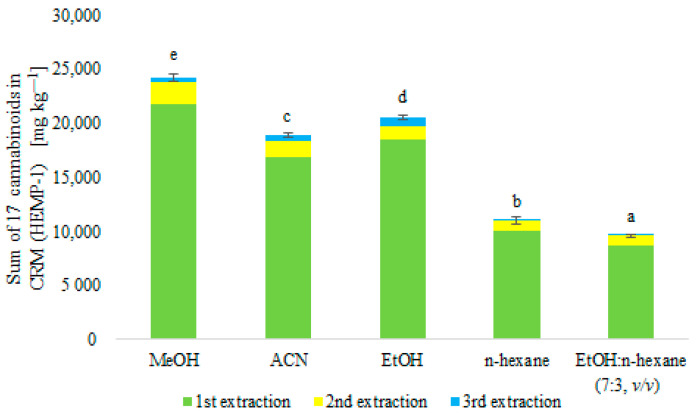
Comparison of the extracted sum of 17 cannabinoids using different solvents from CRM (HEMP-1). a–e—symbol indicates a statistically significant result; *n* = 3.

The extraction efficiency of the sum of 17 cannabinoids from the control CRM-dried material (HEMP-1) was compared using different extraction solvents (MeOH, ACN, EtOH, n-hexane, and EtOH:n-hexane mixture (7:3, *v*/*v*) (Figure 2). During the analyses, Δ^8^-THC was found to be present in the CRM even though there was no specific certified value for this compound. In the results presented here, this compound was taken into account when comparing the extraction efficiency of the sum of 17 cannabinoids from the CRM. A three-step extraction was performed using each extraction solvent (10 mL), and the sum of 17 cannabinoids extracted at each extraction step was compared (Figure 2). In the case of using n-hexane and a mixture of EtOH:n-hexane (7:3, *v*/*v*), the obtained recovery results of 69% and 60%, respectively, were the lowest in terms of the extracted sum of cannabinoids. Based on the results obtained, these two types of solvents were discarded. Performing the first stage of extraction allowed the extraction efficiency of 90% (MeOH), 69% (ACN), and 77% (EtOH) of the sum of 17 cannabinoids present in the CRM. The second extraction step yielded 8% (MeOH), 6% (ACN), and 5% (EtOH) of the cannabinoids, while the third extraction step yielded only 2% (MeOH and ACN) and 3% (EtOH).

The results showed statistically significant differences (*p* < 0.01) between the contents of the tested compounds in MeOH, ACN, and EtOH extracts (Figure 2). Based on the results, it was concluded that MeOH was the most suitable extraction liquid and that the two-stage extraction provided a satisfactory sum of the extracted cannabinoid content.

### 2.2. Optimization of the Cannabinoid Analysis Process Using UHPLC-HESI-MS

**Figure 3:**
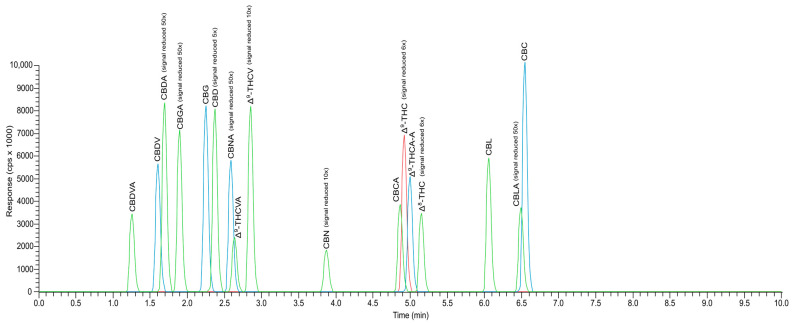
UHPLC-HESI-MS chromatogram of 17 cannabinoids in a calibration standard concentration of 10 μg mL^−1^ for each cannabinoid.

Several authors have performed chromatographic separation using mass spectrometry [12,16,17]. Optimization of the mass spectrometer operation (voltage on the capillary, gas flow) was realized based on the analysis of variants of the values of these parameters (unpublished data), and the results of this experiment are presented in Section 4.5. Using a high-resolution mass spectrometer made it possible to achieve better sensitivity of the method and obtain very low quantification limits. The sample’s high dilution, described in Section 4.4, indicates that no secondary fragmentation of compounds is required during the analysis. Most of the published work has analyzed fewer cannabinoids than in our work [12,18]. There are first reports of analysis of 17 cannabinoids or more [16,17]. In the studies presented here, the separation of analytes was evaluated using variants presented in the above works. The authors used, among others, water and/or ACN with 0.1% formic acid as mobile phases [12,16,17]. The analyses conducted by the above authors were operated in a mobile phase gradient. In our work, analyses were carried out using the isocratic elution method. Given that several cannabinoids were characterized by the isomerism of these compounds and, as a result, identical molecular weights, chromatographic separation of the tested substances was an important issue. Identification of the compounds in the tested samples was carried out by comparing their retention times and mass spectra with analytical standards for these compounds. Experimentally, it was shown that the most effective mobile phase for the separation of analytes mixture of ACN: 0.02% HCOOH_aq_ and 5 mM HCO_2_NH_4 aq_ (75:25, *v*/*v*), which enabled separation in the shortest possible time (10 min). This is one of the first methods to allow analysis of cannabinoids in such a short time. The chromatogram for all 17 cannabinoids analyzed in this study is shown in Figure 3.

The retention time and precursor ions for the compound are shown in Table 1.

### 2.3. Method Validation

Due to the lack of cannabinoid-free matrix availability, adding these compounds or their internal standards directly to the matrix before extraction is not practiced. Internal standards are most often added to diluted extracts at the final stage of sample preparation [16]. An important element of method validation in the determination of cannabinoids in samples is the determination of the matrix effect. The absolute matrix effect was calculated by comparing the slope of the matched-matrix standard curve with the slope of the standard calibration curve. The matrix effect values obtained for all matrices (inflorescences, leaves, and dried ground hemp) ranged from 97 to 106% (Table 2), and it can be concluded that there is no matrix effect on the final assay result.

To prepare calibration curves of the test substances, solutions of these compounds were prepared. The linearity of each of the analyzed compounds was evaluated based on eight-point calibration curves determined by analyzing standard solutions of different concentration ranges. These ranges were chosen to consider the different levels of cannabinoid content in the plant. The concentration-response relationship of the present method indicated a linear relationship between the concentration and peak area with R^2^ values of >0.995 for all 17 cannabinoids. The LOD (limit of detection) and LOQ (limit of quantification) were evaluated by measuring the response at a signal-to-noise ratio (S/N) of ≥3 for LOD and ≥10 for LOQ, respectively, in all types of samples spiked with the standard solution. The determined LOD and LOQ values (Table 2) were at an appropriate level, which enabled the identification and quantification of compounds with low concentrations in inflorescences, leaves, and dried hemp material. The LOQ tended to be much lower than the range of the calibration curves since the analyzed compounds did not occur at such low levels; nevertheless, the low LOQ values allow for future analysis of these compounds at much lower levels. The range of calibration curves, coefficients of determination, and LOD and LOQ for each substance are shown in Table 2. According to the AOAC [19], the concentration of added analyte should be no less than the initial concentration, and the fortified test sample’s response must not exceed the calibration curve’s highest point [19]. The general assumptions of AOAC [19] and ICH [20] for the validation of analytical methods are that the recovery values of the analytes tested should be in the range of 80 to 120%, while the RSD for recovery must not exceed 15%. Recovery analyses were performed for all materials used: fresh inflorescences, leaves, and dried ground hemp (CRM). The recovery and repeatability results of the method obtained in our case meet these criteria. In the case of inflorescences, leaves, and CRM, the recovery values ranged from 97 to 100%, 94% to 101%, and 94 to 103% (depending on the type of compound), respectively, while the RSD value was not greater than 10% (Supplementary Materials Table S1). Most authors report LOQ values in µg kg^−1^, but in our work, this parameter is expressed in μg mL^−1^ due to the wide range of dilutions used, which affect the final LOQ for a given compound. The LOQ values obtained by Brighenti et al. [12] ranged from 1.8 to 2.5 μg mL^−1^, depending on the substance. A lower LOQ (1 μg mL^−1^) was characterized by the method developed by Zivovinovic et al. [6]. De Becker et al. [8] developed a determination method whose LOQs for the analyzed compounds were in the range of 0.06–0.25 μg mL^−1^. These values are much higher than those obtained in our work, which allows us to conclude that the present one allows the determination of cannabinoids at much lower concentrations. Differences in the LOQs obtained for the different methods depend on the technique used for cannabinoid analysis.

#### Method Validity: Analysis of CRM and Material for the Proficiency Test

The correctness of the method for the determination of cannabinoids was evaluated on the basis of the analysis of certified reference material. Extractions were carried out according to the optimized procedure for dried material described in Section 4.4. The correctness of the method was assessed by analyzing the cannabinoid content of the certified reference material (HEMP-1 CRM—dried ground hemp). This evaluation was performed according to the recommendations of the Institute for Reference Materials and Measurements (IRMM-JRC) [21]. This evaluation consisted of checking whether the absolute difference between the determined analyte content and the certified value was significantly smaller than the composite expanded measurement uncertainty and the uncertainty of the certified value (Table 3). For this purpose, it was necessary to determine the expanded uncertainty of the method, which took into account all relevant sources of uncertainty in the analytical procedure for the determination of cannabinoids (data not presented). The uncertainty of the method was evaluated according to EURACHEM/CITAC guidelines [22]. The composite expanded uncertainty was calculated using the following equation (Equation (1)):

(1) $$u=\sqrt{{\left(0.5U\right)}^{2}+{\left({0.5U}_{CRM}\right)}^{2}};\;U\;=\;2u$$

where u is the composite uncertainty, U is the composite expansion intensity, and U_CRM_ is the certified concentration value.

The results obtained for the proficiency tests for dried hemp provided by ASTM International (HFL2301 and HFL2305) were within the accepted criteria and are the basis for confirming the validity of the developed method (Supplementary Materials Tables S2 and S3).

### 2.4. Application of the Method to the Determination of Cannabinoids in Different Samples from Cannabis sativa L. var. sativa

#### 2.4.1. Analysis of the Content of 17 Cannabinoids in the Fresh Plant *Cannabis sativa* L. *var. sativa* ‘Białobrzeskie’

Using the developed and validated method, the cannabinoid content of inflorescence and leaf samples (*n* = nine samples for each part from nine different plants) was evaluated. The results of the content of each of the 17 cannabinoids are presented in Table 4. The research material used in the study, i.e., *Cannabis sativa* L. *var. sativa* “Bialobrzeskie” is a plant that, on a plant dry weight basis, can contain a maximum of 0.3% Δ^9^-THC. The plant is known for its high CBD content and is used on an industrial scale to produce cannabinoid-containing foods [23,24].

The realized research made it possible to conclude that the level of some cannabinoids in different parts of the plant varied. The highest total content of the tested compounds was found in small inflorescences (11,993 mg kg^−1^), while the lowest was found in leaves (3004 mg kg^−1^). It was found that samples of big inflorescences had a statistically significant (*p* < 0.01) lower sum of 17 cannabinoids than that determined in small and medium inflorescences. Analyzing individual substances, the highest contents were recorded for CBDA, which was the dominant cannabinoid in all elements of the plant. Samples of small inflorescences had significantly (*p* < 0.01) higher contents of Δ^9^-THCA-A and CBCA. The inflorescences and leaves showed no evidence (<LOD) of cannabinoids such as CBL, CBN, and Δ^8^-THC. There were no statistically significant differences (*p* < 0.01) in the content of CBD, CBDA, CBLA, CBGA, and CBDV in inflorescences of different sizes. Based on the analyses, the leaves were found to have a significantly (*p* < 0.01) higher content of cannabinoids such as CBD, CBGA, CBLA, and CBNA compared to all analyzed inflorescences. When analyzing the cannabinoids in the plant, it is also important to consider the total Δ^9^-THC content and total CBD content, which indicates the total content of acid and neutral forms of Δ^9^-THC and CBD. Statistically significant differences (*p* < 0.01) were found in total Δ^9^-THC content and total CBD content in both inflorescences and leaves.

#### 2.4.2. Analysis of Cannabinoid Content in Teas Based on *Cannabis sativa* L. *var. sativa*

The objective of this study was also to analyze the content of cannabinoids in commercially available teas based on *Cannabis sativa* L. *var. sativa*. Thirty samples of teas containing 40 to 100% dried hemp in their composition were analyzed. The results of the content of these compounds in each sample are shown in Table 5 and Supplementary Materials Table S4.

The realized research made it possible to conclude that the level of content of some cannabinoids in the analyzed teas varied greatly. The highest total content of 17 cannabinoids was recorded in tea No. 30 (60% dried hemp—19,723 mg kg^−1^), while the lowest was recorded in tea No. 20 (75% dried hemp—2962 mg kg^−1^). Statistically significant differences (*p* < 0.01) were found between the total content of the 17 cannabinoids in the analyzed teas. Taking into account the percentage of dried hemp in the analyzed teas, it is not possible to unequivocally conclude that products containing 100% dried hemp contain more cannabinoids compared to teas with a lower percentage of dried hemp. Analyzing individual substances, the highest contents were recorded for CBDA (501.2–11,692 mg kg^−1^), CBD (308.0–6448 mg kg^−1^), and CBGA (33.3–3646 mg kg^−1^). Δ^9^-THCA-A, which is the precursor for Δ^9^-THC, was present in the tested samples in contents ranging from 58.3 to 948.7 mg kg^−1^. Δ^9^-THC in the teas analyzed fell within a fairly wide range of content, i.e., from 21.4 to 616.8 mg kg^−1^, but did not exceed the permissible limit of 0.3% in dry plant material [25]. CBN (4.3–80.0 mg kg^−1^), Δ^8^-THC (2.5–8.5 mg kg^−1^), and Δ^9^-THC exhibit psychoactive effects, so it is extremely important to control the content of food products, including teas based on *Cannabis sativa* L. *var. sativa*. A comparison of total Δ^9^-THC content and total CBD content in 30 tea samples was also made. The results are shown in Table 5 and Table S4 (Supplementary Materials). Performing the analysis of total Δ^9^-THC content and total CBD content allows determining the total content of neutral and acid forms of Δ^9^-THC and CBD in the plant. Statistically significant differences (*p* < 0.01) were found in the total Δ^9^-THC content and total CBD content of the analyzed teas. In none of the analyzed tea samples was the total content of Δ^9^-THC greater than 0.3% of the dry weight of the *Cannabis sativa* L. *var. sativa* plant (0.01–0.09% of dry weight). The total CBD content ranged from 747.6 to 13,686 mg kg^−1^ (0.08–1.37% of dry weight).

## 3. Discussion

### 3.1. Optimization of the Procedure for the Extraction of Cannabinoids from the Tested Matrices

#### 3.1.1. Fresh Parts of Plants

There is little information regarding the extraction of cannabinoids from fresh *Cannabis sativa* L. *var. sativa* plants. Most studies have already analyzed cannabinoids in dried plants. Our results are among the first regarding the comparison of cannabinoid content in different parts of the fresh plant, taking into account inflorescence size. Namdar et al. [26] extracted cannabinoids from fresh inflorescences of *Cannabis sativa* L. using EtOH, n-hexane, and a mixture of EtOH:n-hexane (7:3 *v*/*v*). The authors showed that it was possible to extract the highest sum of the analyzed cannabinoids using a mixture of EtOH:n-hexane (7:3, *v*/*v*). The same authors also showed differences in the content of cannabinoids in inflorescences occurring in the upper part of the plant and the lower parts. The study showed that significantly more cannabinoids were found in the top inflorescences, which were also the biggest in the plant. The results obtained by Namdar et al. [26] do not coincide with those obtained in our study, where it was shown that the best extraction liquid is MeOH rather than a mixture of EtOH:n-hexane (7:3, *v*/*v*). Our study also showed that small inflorescences contained more cannabinoids than big inflorescences. The differences may be due to a different extraction method, a different plant variety, different growing conditions, a different degree of maturity of the plant at harvest, and different inflorescence structures (higher proportion of leaves/stalk) [26].

#### 3.1.2. Dried Plants

For practical reasons, to evaluate the degree of extraction with the tested solvents, dried ground hemp was used as a certified reference material; this is of particular importance since, on this basis, it is possible to assume a certain sum of the tested compounds. In the literature, one can find many described extraction methods that are based on the use of mainly solvent mixtures. Not every case cited evaluated the same extraction liquids for extracting cannabinoids as in the study presented by us. Hence, it is difficult to have a fair discussion. Zivovinovic et al. [6] compared different solvent mixtures for extracting cannabinoids from *Cannabis sativa* L. inflorescences. They used mixtures of MeOH:H_2_O (4:1, *v*/*v*), MeOH:CHCl_3_ (9:1, *v*/*v*), ACN:H_2_O (1:1, *v*/*v*) and 100% ACN for extraction. Extraction with ACN yielded the lowest sum of extracted cannabinoid content. A similar observation was observed in our study. The other mixtures used by the authors had similar cannabinoid extraction yields, but for further analysis, the authors chose the ACN:H_2_O (1:1, *v*/*v*) mixture for practical reasons [6]. A study by Mudge et al. [13] found that using a mixture of MeOH: H_2_O (8:2, *v*/*v*) yields a similar sum of extracted cannabinoids from the plant compared to a mixture of MeOH:CHCl_3_ (9:1, *v*/*v*) [13]. McRae and Melanson [16] analyzed the efficiency of a five-step extraction of cannabinoids from dried *Cannabis sativa* L. material using a mixture of MeOH:H_2_O (8:2, *v*/*v*) and MeOH:CHCl_3_ (9:1, *v*/*v*). They showed that with these mixtures, more than 98% extraction efficiency could be achieved by two-stage extraction. The results obtained by the author confirm that the use of MeOH is legitimate in the extraction of cannabinoids. In the present study, such mixtures were not used because cannabinoids are water-insoluble compounds, and the use of CHCl_3_ during the extraction of these compounds, as demonstrated by McRae and Melanson [16], does not increase extraction efficiency, as this liquid is an extremely volatile substance and exhibits high toxicity [6,16].

### 3.2. Application of the Method to the Determination of Cannabinoids in Different Samples from Cannabis sativa L. var. sativa

#### 3.2.1. Analysis of the Content of 17 Cannabinoids in the Fresh Plant *Cannabis sativa* L. *var. sativa* ‘Białobrzeskie’

To date, only a few cases in the literature have presented the distribution of cannabinoids in different parts of the plant [27,28,29]. Most authors focus mainly on analyzing a few selected cannabinoids. Kleinhenz et al. [28] showed a total cannabinoid content in inflorescences and leaves of 46,076 and 52,021 mg kg^−1^, respectively. Different results were obtained in our study, where the contents of the analyzed substances were at a much lower level. The obtained differences in the total content may be due to the different degrees of maturity of the plant, since, with the maturation of the inflorescences, the contents of cannabinoids change. It should be mentioned that our work obtained differences in the contents of some cannabinoids between different parts of the plant. Kleinhenz et al. [28] recorded the highest concentration of CBGA in inflorescences at 1938 mg kg^−1^. In our study, the content of this compound in the analyzed inflorescences of *Cannabis sativa* L. *var. sativa* “Białobrzeskie” plant was approximately ten times lower, averaging 164.3 mg kg^−1^. Only in the case of CBD did Kleinhenz et al. [28] show no difference in the content of this compound between inflorescences and leaves [28]. Our study showed more than twice the concentration of CBD in leaves (168.7 mg kg^−1^) than in medium-sized inflorescences (65.7 mg kg^−1^). Kalinova et al. [29] determined the contents of CBD, CBDA, and CBGA in the inflorescences and leaves of the two most popular varieties of *Cannabis sativa* L. *var. sativa* “Białobrzeskie” and “Finola”. In the inflorescences and leaves of the “Białobrzeskie” variety, more CBD (inflorescences 262.9 mg kg^−1^, leaves 56.7 mg kg^−1^) and CBDA (inflorescences 11,519 mg kg^−1^, leaves 4267 mg kg^−1^) were determined compared to the “Finola” variety, which contained 135.4 mg kg^−1^ (inflorescences), 16.54 mg kg^−1^ (leaves) CBD and 8707 mg kg^−1^ (inflorescences), 1792 mg kg^−1^ (leaves) CBDA, respectively. The “Finola” variety had a higher CBGA content in inflorescences (1345 mg kg^−1^) than the “Białobrzeskie” variety (905.4 mg kg^−1^) [29]. The CBDA and CBD contents determined by the authors [29] for the inflorescences and leaves were significantly higher than those determined in our study for the same plant variety. Ibrahim et al. [27] also showed differences in CBD and CBDA contents in inflorescences and leaves of *Cannabis sativa* L. *var. sativa* fiber hemp. The contents were in the range of 13,100–21,100 mg kg^−1^ (inflorescences) and 5700–23,000 mg kg^−1^ (leaves) for CBDA and of 1000–3900 mg kg^−1^ (inflorescences) and 700–18,600 mg kg^−1^ (leaves) for CBD. The above study also showed a higher content of CBD and CBDA in inflorescences and leaves extracted from fiber hemp plants than in the research material used in this article. The indicated differences in the content of cannabinoids in different parts of the plant in our article and the cited works may be influenced by several reasons. One of them is the lack of standardized testing methods through which different authors can obtain different results when analyzing plants of the same variety. Another reason is the degree of maturity of the inflorescences and the whole plant. According to Aizpurua-Olaizola et al. [30], the content of major cannabinoids such as CBDA, Δ^9^-THCA-A, and CBGA changes with the maturation of the plant in both inflorescences and leaves. Therefore, when analyzing cannabinoid content, it is important to provide data on when the plant was harvested. As Eržen et al. [31] and Fischedick et al. [32] confirmed, growing conditions and geographic location also affect the plant’s cannabinoid content. Droughts or excessive rainfall can induce stress conditions in plants, which can synthesize cannabinoids to varying degrees. Park et al. [33] reported that in early flowering, drought stress is the main cause of changes in the cannabinoid profile in inflorescences, altering cannabinoid production by reducing CBD and Δ^9^-THC accumulation and increasing CBG accumulation by 40%. Other authors suggest that the plant’s production and accumulation of cannabinoids are also affected by light intensity, ambient temperature, the presence of nutrients, high concentrations of heavy metals in the soil, phytohormones, and stresses caused by insect and microbial pathogens [33,34,35,36]. Gorelick and Bernstein [36] showed that both supplementation with selected minerals and their deficiency can affect the plant’s cannabinoid profile.

#### 3.2.2. Analysis of Cannabinoid Content in Teas Based on *Cannabis sativa* L. *var. sativa*

Currently, there are few studies analyzing the cannabinoid content of *Cannabis sativa* L.-based hemp teas or dries as raw material for hemp teas. The few existing ones present varying levels of cannabinoid content in these products [37,38,39]. Knezevic et al. [38] determined the content of selected cannabinoids (CBDA, CBD, Δ^9^-THCA-A, Δ^9^-THC, CBN) in hemp-leaf-based teas. The results ranged from 4.1 mg kg^−1^ CBDA, 802.0 mg kg^−1^ CBD, 111.0 mg kg^−1^ Δ^9^-THCA-A, 76.0 mg kg^−1^ Δ^9^-THC, and 52.0 mg kg^−1^ CBN. The values obtained for hemp-leaf-based teas in the cited work were within the range of occurrence of the analyzed compounds analyzed in our work. Hemp-leaf-based teas contained significantly less Δ^9^-THC than those containing inflorescences [38]. In the study by Kladar et al. [39], the hemp teas analyzed contained both hemp leaves and inflorescences. The determined total Δ^9^-THC content for the tea samples (dry herbal material) ranged from 13 to 8831 mg kg^−1^. It should be noted that one of the samples analyzed by Kladar et al. [39] contained almost 0.9% total Δ^9^-THC. This value is above the limit set by the EC (EC Regulation 2021/2115), which is 0.3% for Δ^9^-THC on a plant dry weight basis [25]. CBD levels in the samples analyzed ranged from 444 to 25,004 mg kg^−1^, and CBN levels ranged from 14 to 266 mg kg^−1^ [39]. The quoted cannabinoid contents of the teas far exceed the levels of selected cannabinoids in the teas we analyzed. In the context of assessing the content of Δ^9^-THC, our results indicate that its content was below 0.3% of dry weight. However, in the literature, these contents were generally higher. Gallo-Molina et al. [10] identified this compound at an average level of 3550 mg kg^−1^ plant, which exceeded the permitted content set by the EU. Pacifici et al. [37] also analyzed hemp teas for cannabinoid content. The authors found the presence of CBDA, CBG, CBD, CBN, Δ^9^-THC, CBC, and Δ^9^-THCA-A in dried tea at levels averaging 61,800, 600, 26,600, 900, 33,700, 1200, and 28,200 mg kg^−1^, respectively. The occurrence of cannabinoids at such a wide level of content in individual teas may be related, among other things, to the additives used in these products, such as herbs or dried fruits, the proportion of which (0–60%) affects the composition of hemp tea. Pure hemp teas analyzed in our study based on dried hemp alone (inflorescences and leaves) tended to have higher cannabinoid content than teas containing 40–100% dried hemp (Table 5). When assessing cannabinoid intake, it is important to consider that teas are made into infusions. Cannabinoids dissolve poorly in water, so incomplete intake of these substances from the product is to be expected. The wide range of cannabinoids in finished products such as dried or hemp teas based on *Cannabis sativa* L. *var. sativa* suggests that it is necessary to control the level and profile of cannabinoids in finished products on the market to exclude possible exceedances of Δ^9^-THC in the product.

## 4. Materials and Methods

### 4.1. Sample Material

*Cannabis sativa* L. *var. sativa* cultivars ‘Bialobrzeskie’ were selected as research material due to its recorded history of human consumption, the widespread use of the plant for CBD extraction, and the production of food containing cannabinoids. The plants were obtained from the Institute of Natural Fibers and Herbaceous Plants in Poznań, Pętkowo, Poland. The plants were harvested at the peak of flowering (between the twentieth day after the start of flowering and the tenth day after the end of flowering) [40]. The harvested plants were divided into inflorescences and leaves, and the remaining plant parts were removed. Inflorescences were divided by size (length) into small (<10 cm), medium (10–20 cm), and big (>20 cm). The samples prepared in this way were then frozen and stored at −60 °C. Thirty samples of *Cannabis sativa* L. *var*. *sativa*-based teas available on the Polish market were also analyzed. Certified reference materials of dried (HEMP-1) ground hemp were purchased from the National Research Council Canada. A CRM was used for validation and quality control purposes. This material has been rigorously tested to be homogeneous and stable concerning the 16 cannabinoids except for Δ^8^-THC, for which the concentration was not determined. In this article, an attempt was made to determine Δ^8^-THC in the CRM analyzed. The material for the proficiency tests was dried hemp provided by ASTM International (HFL2301 and HFL2305) (Supplementary Materials Tables S2 and S3).

### 4.2. Chemicals and Reagents

The certified reference materials of cannabidiol (CBD), cannabidiolic acid (CBDA), cannabigerol (CBG), cannabichromene (CBC), cannabinol (CBN), cannabidiolic acid (CBNA), cannabidivarinic acid (CBDVA), cannabicyclol (CBL), and cannabicyclic acid (CBLA) were provided at 1.0 mg mL^−1^ solutions in methanol (MeOH) or acetonitrile (ACN) from Restek GmbH (Bad Homburg, Germany). Cannabigerolic acid (CBGA), cannabichromenic acid (CBCA), Δ^9^-tetrahydrocannabinol (Δ^9^-THC), Δ^8^-tetrahydrocannabinol (Δ^8^-THC), Δ^9^-tetrahydrocannabinolic acid (Δ^9^-THCA-A), Δ^9^-tetrahydrocannabivarinic acid (Δ^9^-THCVA), and cannabidivarin (CBDV) were purchased from LGC Standards. Δ^9^-tetrahydrocannabivarin (Δ^9^-THCV) was provided at 1.0 mg mL^−1^ solutions in MeOH or ACN from Cerilliant Corporation (Round Rock, TX, USA). The certified purity value for all the CRMs was >98.00%. Liquid chromatography–mass spectrometry (LC-MS)-grade water, ACN, MeOH, ethanol (EtOH), and n-hexane were purchased from Witko (Lodz, Poland). Formic acid and ammonium formate (LC-MS grade) were obtained from Sigma-Aldrich (St. Louis, MO, USA).

### 4.3. Preparation of Standard Solutions and Calibration Curves

Standard 100 μg mL^−1^ solutions for all 17 cannabinoids were prepared by dissolving 1.0 mL of the compound reference standard in ACN or MeOH using 10 mL volumetric flasks separately. This step was repeated as it was necessary to prepare higher dilutions for most compounds except CBD and CBDA. All solutions were stored at <−80 °C. Eight-point curves were prepared for 17 cannabinoids in different ranges, which were generated using Thermo TraceFinderTM software, version 5.1 (Thermo Fisher Scientific, Pleasanton, CA, USA). Curves in matrix were prepared by adding specific amounts of the standard (as in the curve in solvent) to a previously prepared and appropriately diluted extract (fresh inflorescence, fresh leaves, CRM).

### 4.4. Preparation of Test Samples

Before starting cannabinoid extraction, all samples previously frozen (−60 °C) were ground to a fine powder using a Grindomix GM200 grinder (Retsch, Haan, Germany). 

Fresh material: 0.1 g was weighed into 50 mL Falcon vial samples of inflorescences or leaves and extracted with MeOH in a volume of 5 mL. 

Dried material (ground dried hemp—CRM and hemp tea): 0.1 g to 50 mL Falcon vial samples were weighed and extracted twice with 10 mL of MeOH. 

Samples were homogenized (2 min. 5000 rpm) using a Unidrive X 1000 homogenizer, Cat SCIENTIFIC (CAT Scientific Inc., Paso Robles, CA, USA). The prepared samples were then centrifuged (2 min. 10,000 rpm) using an MPW-380R centrifuge from MPW Med. Instruments (Warsaw, Poland). The obtained extracts of the dried material after extraction were mixed. For analyses, 1 mL of extract filtered through a 0.22 µm 13 mm syringe filter was used (LLG Labware, Meckenheim, Germany). For compounds for which concentrations outside the upper range defined by the calibration curve were observed, the extracts were diluted accordingly with the extraction liquid. Dry weight content was determined using a WPS 30S balance dryer (Radwag, Radom, Poland) to express cannabinoid levels relative to the dry mass. The level of the studied cannabinoids was calculated according to the following formula (Equation (2)):

C = a/b(2)

where: C—the content of the studied compound (in µg kg^−1^) calculated per dry weight; a—determined content of the studied compound (in µg kg^−1^); b—dry weight content (in % of the fresh weight content).

### 4.5. UHPLC-HESI-MS

Cannabinoids were analyzed using ultra-high-performance liquid chromatography-Q-Exactive Orbitrap mass spectrometry setup operating with a heated electrospray interface (UHPLC-HESI-MS) (Thermo Fisher Scientific, Waltham, MA, USA). Analyses were performed on a 2.1 × 100 mm, C18 Cortecs, 1.6 µm chromatography column (Waters, Milford, MA, USA). The mobile phase consisted of a mixture of ACN: 0.02% HCOOH_aq_ and 5 mM HCO_2_NH_4 aq_ (75:25, *v*/*v*). Elution of analytes was carried out in isocratic mode. A constant flow rate of 0.3 mL min^−1^ (10 min) and a constant injection volume of 2 µL were used for all analyses. The column thermostat temperature was 20 °C. During ionization in the mass spectrometer, the heated electrospray ionization (HESI) mode of operation was used in both positive and negative polarizations with a scan range of 100–1000 *m*/*z*. The parameters of the ionization source were as follows: spray gas flow rate (nitrogen): 50 L h^−1^; auxiliary gas flow rate (nitrogen): 1 L h^−1^; spray voltage: +2300 V and −2000 V; capillary temperature: 305 °C.

### 4.6. Method Validation

The method validation was based on AOAC International guidelines [19] and by the Validation of Analytical Procedures: Text and Methodology Q2 to meet the requirements of the International Council for Harmonization [20]. The proposed analytical method was validated for linearity, limits of detection (LOD) and quantification (LOQ), recovery (R), and precision (RSD). Matrix effects were measured by comparing the ratio of the slope coefficient of the calibration curve prepared from the analysis of standards dissolved in a solvent to the slope of the same calibration curve resulting from the study of these standards in the all-tested matrix. A value of 100% indicates that there is no matrix effect. There is signal amplification if the values are >100% and signal attenuation if the values are <100%. The extraction recovery was measured by comparing the peak area of all analyzed matrix spiked with standards before and after extraction.

### 4.7. Statistical Analyses

Statistical results were analyzed using Statistica 13.1 software (Statsoft, Carlsbad, CA, USA). One-way variance analysis (one-way ANOVA) was used to determine the significant differences between the contents of cannabinoids in plant material. The significance of differences was determined at a significance level of α = 0.01. The homogeneity of the groups was determined using the Tukey HSD test.

## 5. Conclusions

This article presents the characterization of a method for determining up to 17 selected cannabinoids in fresh hemp (inflorescences and leaves) and dried material (CRM and hemp teas). This is one of the first works on the analysis of 17 cannabinoids in such matrices using a 2.1 × 100 mm, C18 Cortecs, 1.6 µm chromatography column (Waters). The developed method was characterized by desirably low LOD and LOQ values. The method showed correct recovery values of individual compounds and inter- and intraday precision and accuracy values. MeOH was found to be the best extraction liquid for cannabinoids in fresh and dried material. It was shown that the dominant cannabinoids in fresh plant parts (inflorescences and leaves) are CBDA, Δ^9^-THCA-A, and CBCA, and in teas containing dried hemp CBDA, CBD, CBGA, and Δ^9^-THCA-A. The presence of psychoactive cannabinoids (Δ^9^-THC, Δ^8^-THC, and CBN) in teas available on the food market, which was confirmed in the samples analyzed, may be a problem for manufacturers. Due to the varying levels of cannabinoids in *Cannabis sativa* L.-based products, further work is needed to develop a standard procedure for extracting these compounds. To date, the levels of permissible contents of total Δ^9^-THC and Δ^9^-THCA-A established by EU law apply only to seeds and products derived from them.

## Figures and Tables

**Table 1:** Retention time and precursor ions for a specific compound in the UHPLC-HESI-MS system.

Compound	Retention Time	Precursor Ion [*m*/*z*]
**CBDVA**	1.25	329.1737 [M − H]^−^
**CBDV**	1.6	287.1992 [M + H]^+^
**CBDA**	1.7	359.2043 [M − H]^−^
**CBGA**	1.9	359.2208 [M − H]^−^
**CBG**	2.2	317.2471 [M + H]^+^
**CBD**	2.4	315.2305 [M + H]^+^
**CBNA**	2.6	353.1732 [M − H]^−^
**Δ^9^-THCVA**	2.65	329.1744 [M − H]^−^
**Δ^9^-THCV**	2.85	287.1993 [M + H]^+^
**CBN**	3.9	311.2003 [M + H]^+^
**CBCA**	4.85	359.2177 [M − H]^−^
**Δ^9^-THC**	4.95	315.2314 [M + H]^+^
**Δ^9^-THCA-A**	5.0	359.2038 [M − H]^−^
**Δ^8^-THC**	5.15	315.2305 [M + H]^+^
**CBL**	6.1	315.2305 [M + H]^+^
**CBLA**	6.5	359.2065 [M − H]^−^
**CBC**	6.6	315.2305 [M + H]^+^

**Table 2:** Range of calibration curves, coefficients of determination, LOD, LOQ, and matrix effect for each of 17 cannabinoids.

Compound	Range of Calibration Curve [µg mL^−1^]	Coefficient of Determination [R^2^]	LOD [μg mL^−1^]	LOQ [μg mL^−1^]	LOD [mg kg^−1^]	LOQ [mg kg^−1^]	Matrix Effect (%)		
							Inflorescences	Leaves	CRM
**CBD**	0.200–25.600	0.9997	0.0010	0.0030	1.8	6	99	101	100
**CBN**	0.008–1.024	0.9991	0.0020	0.0070	1.2	4	99	99	99
**CBG**	0.008–1.024	0.9997	0.0003	0.0009	0.6	2	99	100	100
**Δ^9^-THC**	0.080–10.240	0.9999	0.0011	0.0036	0.6	2	97	99	97
**Δ^8^-THC**	0.008–1.024	0.9993	0.0020	0.0060	0.48	1.6	100	100	104
**CBC**	0.020–2.560	0.9993	0.0050	0.0200	0.6	2	100	99	107
**CBL**	0.020–2.560	0.9991	0.0020	0.0070	0.6	2	100	100	101
**CBDV**	0.008–1.024	0.9994	0.0010	0.0020	0.12	0.4	101	100	101
**Δ^9^-THCV**	0.008–1.024	0.9982	0.0006	0.0020	0.06	0.2	99	100	99
**CBDA**	0.400–51.200	0.9950	0.0003	0.0009	5.4	18	102	100	106
**CBNA**	0.002–0.256	0.9947	0.0001	0.0002	0.12	0.4	101	101	106
**CBGA**	0.020–2.560	0.9967	0.00003	0.0001	0.06	0.2	101	99	101
**Δ^9^-THCA-A**	0.080–10.240	0.9972	0.0004	0.0013	0.6	2	100	100	99
**CBCA**	0.080–10.240	0.9983	0.0008	0.0030	0.6	2	100	100	102
**CBLA**	0.020–2.560	0.9954	0.0004	0.0012	0.06	0.2	100	100	100
**CBDVA**	0.020–2.560	0.9954	0.00003	0.0001	0.06	0.2	98	99	98
**Δ^9^-THCVA**	0.020–2.560	0.9974	0.0001	0.0002	0.01	0.04	99	99	97

**Table 3:** Results of the determination of cannabinoids in certified reference material with the declared concentration value.

Compound	Declared Values		Determined Values		
	C_CRM_ [mg kg^−1^]	U_CRM_ [mg kg^−1^]	C [mg kg^−1^]	U [mg kg^−1^]	*n*
**CBC**	325.0	84.0	321.8	25.8	9
**CBDV**	188.0	32.0	187.3	15.0	9
**CBG**	47.8	9.4	47.3	3.3	9
**CBL**	74.1	13.6	74.0	5.2	9
**CBN**	490.0	70.0	484.2	38.7	9
**CBNA**	350.0	36.0	344.9	27.6	9
**Δ^8^-THC**	- ^1^	-	21.5	1.9	9
**Δ^9^-THC**	318.0	86.0	319.4	28.7	9
**Δ^9^-THCV**	14.3	2.0	14.1	1.3	9
**CBDVA**	719.0	54.0	714.8	64.3	9
**CBD**	5410.0	700.0	5398.7	377.9	9
**CBGA**	117.0	12.0	113.5	9.1	9
**Δ^9^-THCVA**	72.8	6.4	72.3	6.5	9
**CBLA**	187.0	18.0	184.5	14.8	9
**CBCA**	448.0	108.0	450.5	40.6	9
**Δ^9^-THCA-A**	979.0	84.0	966.5	87.0	9
**CBDA**	14,600	800.0	14,536	1308	9
Total Δ^9^-THC content *	1180	140.0	1167	105.0	9
Total CBD content **	18,200	1200	18,147	1633	9

C_CRM_—certified concentration value; C—determined value concentration; U_CRM_—declared composite expanded uncertainty (k = 2); U—designated expanded compound uncertainty (k = 2); *n*—number of independent repeats; ^1^—no certified Δ^8^-THC content in the CRM material analyzed; * total Δ^9^-THC content: Δ^9^-THC + (0.877 × Δ^9^-THCA-A); ** total CBD content: CBD + (0.877 × CBDA) [3].

**Table 4:** Content of cannabinoids in *Cannabis sativa* L. *var. sativa* “Białobrzeskie”. The results of the contents of the individual 17 cannabinoids are presented on a dry weight basis.

Compound	Part of the Plant [mg kg^−1^]			
	Small Inflorescences	Medium Inflorescences	Big Inflorescences	Leaves
**CBC**	16.8 ^c^ ± 1.2	12.4 ^b^ ± 0.6	10.4 ^b^ ± 1.3	3.2 ^a^ ± 0.2
**CBDV**	0.7 ^ab^ ± 0.1	0.4 ^a^ ± 0.1	0.7 ^ab^ ± 0.1	0.8 ^b^ ± 0.1
**CBG**	25.1 ^b^ ± 0.7	40.4 ^c^ ± 1.1	43.2 ^c^ ± 4.1	5.8 ^a^ ± 0.3
**CBL**	<LOD	<LOD	<LOD	<LOD
**CBN**	<LOD	<LOD	<LOD	<LOD
**CBNA**	2.3 ^a^ ± 0.2	3.4 ^b^ ± 0.1	2.4 ^a^ ± 0.2	4.4 ^c^ ± 0.2
**Δ^8^-THC**	<LOD	<LOD	<LOD	<LOD
**Δ^9^-THC**	11.6 ^b^ ± 0.7	16.6 ^c^ ± 0.5	5.9 ^a^ ± 0.5	6.0 ^a^ ± 0.3
**Δ^9^-THCV**	0.2 ^a^ ± 0.1	0.2 ^a^ ± 0.1	0.3 ^a^ ± 0.1	5.9 ^b^ ± 0.2
**CBDVA**	721.3 ^c^ ± 79.8	665.2 ^c^ ± 70.7	385.7 ^b^ ± 37.7	92.1 ^a^ ± 1.4
**CBD**	61.1 ^a^ ± 2.6	65.7 ^a^ ± 7.4	44.5 ^a^ ± 1.5	168.7 ^b^ ± 23.2
**CBGA**	152.5 ^a^ ± 16.4	189.6 ^a^ ± 26.3	150.9 ^a^ ± 12.5	171.6 ^a^ ± 12.2
**Δ^9^-THCVA**	47.9 ^c^ ± 6.9	26.9 ^b^ ± 1.2	25.2 ^b^ ± 2.7	6.1 ^a^ ± 0.5
**CBLA**	17.7 ^a^ ± 2.7	17.5 ^a^ ± 0.9	14.7 ^a^ ± 1.5	31.8 ^b^ ± 0.5
**CBCA**	1371 ^d^ ± 87.7	592.2 ^b^ ± 28.4	818.4 ^c^ ± 74.9	276.3 ^a^ ± 17.1
**Δ^9^-THCA-A**	1418 ^d^ ± 87.6	620.2 ^b^ ± 22.9	842.8 ^c^ ± 77.1	239.9 ^a^ ± 16.5
**CBDA**	8146 ^b^ ± 305.9	9047 ^b^ ± 1347.3	6122 ^b^ ± 863.8	1992 ^a^ ± 285.5
**Total** Δ^9^-THC content *	1255.3 ^d^ ± 44.2	560.6 ^b^ ± 11.7	745.1 ^c^ ± 38.8	216.4 ^a^ ± 8.4
Total CBD content **	7205 ^c^ ± 154.3	8000 ^c^ ± 677.5	5413 ^b^ ± 432.7	1915 ^a^ ± 154.4
**The average sum of 17 cannabinoids**	11,993 ^c^ ± 259.5	11,298 ^c^ ± 303.9	8447 ^b^ ± 675.8	3004 ^a^ ± 240.4

* total Δ^9^-THC content: Δ^9^-THC + (0.877 × Δ^9^-THCA-A); ** total CBD content: CBD + (0.877 × CBDA) [3]; a–d—values within lines followed by the same letter are not significantly different according to α = 0.01.

**Table 5:** The percentage content of the *Cannabis sativa* L. *var. sativa* and the sum of 17 cannabinoid content determined, and the total content of Δ^9^-THC and CBD in the tea samples (mg kg^−1^).

Sample	Percentage Content of *Cannabis sativa* L. *var. sativa*	The Average Sum of 17 Cannabinoids	The Total Δ^9^-THC Content *	The Total CBD Content **
1	100%	15,680 ^ghi^ ± 1254	372.3 ^e^ ± 6.5	13,084 ^p^ ± 21
2	100%	13,769 ^fghi^ ± 1101	514.5 ^j^ ± 5.4	10,852 ^kl^ ± 20
3	100%	9823 ^cde^ ± 785	100.4 ^a^ ± 5.1	3205 ^d^ ± 10
4	100%	13,282 ^efgh^ ± 1062	517.4 ^j^ ± 2.4	8998 ^g^ ± 14
5	80%	15,521 ^ghi^ ± 1241	984.9 ^t^ ± 5.0	12,382 ^o^ ± 100
6	75%	14,317 ^fghi^ ± 1145	273.7 ^c^ ± 10.0	10,938 ^klm^ ± 20
7	75%	17,513 ^ij^ ± 1401	724.1 ^pq^ ± 1.3	13,686 ^q^ ± 233
8	75%	13,461 ^efgh^ ± 1076	650.4 ^m^ ± 2.8	10,659 ^jk^ ± 118
9	75%	18,181 ^ghi^ ± 1454	716.6 ^pq^ ± 5.7	13,551 ^q^ ± 61
10	40%	10,678 ^cdefghi^ ± 854	617.0 ^l^ ± 4.9	7320 ^e^ ± 57
11	100%	13,866 ^fghi^ ± 1109	712.7 ^pq^ ± 5.7	11,054 ^lmn^ ± 60
12	70%	8876 ^bc^ ± 710	447.0 ^h^ ± 1.5	7103 ^e^ ± 70
13	70%	8946 ^bc^ ± 715	436.3 ^h^ ± 5.6	7220 ^e^ ± 53
14	70%	16,845 ^hij^ ± 1281	951.2 ^r^ ± 5.7	9726 ^i^ ± 161
15	70%	9416 ^bcd^ ± 753	492.8 ^i^ ± 1.8	7383 ^e^ ± 81
16	70%	12,255 ^cdefgh^ ± 980	641.5 ^m^ ± 1.6	7176 ^e^ ± 253
17	100%	5960 ^ab^ ± 476.8	193.3 ^b^ ± 3.6	747.6 ^a^ ± 17
18	100%	4107 ^a^ ± 328.6	399.1 ^g^ ± 5.6	2922 ^b^ ± 74
19	100%	12,698 ^cdefgh^ ± 1015	504.4 ^ij^ ± 2.8	10,437 ^jk^ ± 92
20	75%	2962 ^a^ ± 236	323.9 ^d^ ± 1.2	2106 ^b^ ± 181
21	75%	3774 ^a^ ± 301	376.1 ^fg^ ± 1.6	2714 ^c^ ± 132
22	100%	15,162 ^ghi^ ± 1213	726.1 ^q^ ± 1.7	12,267 ^o^ ± 151
23	70%	9506 ^cdef^ ± 760	389.2 ^f^ ± 1.1	7922 ^f^ ± 86
24	100%	13,811 ^fghi^ ± 1104	552.4 ^k^ ± 2.9	11,704 ^n^ ± 224
25	100%	13,102 ^defgh^ ± 1048	625.0 ^l^ ± 2.5	9619 ^hi^ ± 83
26	100%	12,084 ^cdefg^ ± 966	554.8 ^k^ ± 2.8	9324 ^gh^ ± 106
27	100%	13,208 ^defgh^ ± 1056	661.9 ^n^ ± 4.6	10,174 ^j^ ± 100
28	100%	14,176 ^fghi^ ± 1134	684.5 ^o^ ± 3.3	11,331 ^mn^ ± 61
29	100%	9673 ^bcde^ ± 773	684.7 ^o^ ± 2.8	7461 ^e^ ± 98
30	60%	19,723 ^j^ ± 1577	707.8 ^p^ ± 1.0	16,703 ^r^ ± 47

* total Δ^9^-THC content: Δ^9^-THC + (0.877 × Δ^9^-THCA-A); ** total CBD content: CBD + (0.877 × CBDA) [3]; a–r values within columns followed by the same letter are not significantly different according to α = 0.01.

## Data Availability

Data are contained within the article and Supplementary Materials.

## Supplementary Materials

The following supporting information can be downloaded at https://www.mdpi.com/article/10.3390/molecules28248008/s1. Table S1: Intra- and inter-day precision data of the determination of cannabinoids in inflorescences, leaves, and CRM (intra-day *n* = 6; inter-day, day = 3, *n* = 3 days × 6 = 18). Table S2: Results obtained, average values, and z-score for dried hemp proficiency tests (HFL2301). Table S3: Results obtained, average values, and z-score for dried hemp proficiency tests (HFL2305). Table S4: The content of 17 cannabinoids for each hemp tea sample (mg kg^−1^).
