# Supplementary material for: The Development, Validation, and Application of a UHPLC-HESI-MS Method for the Determination of 17 Cannabinoids in Cannabis sativa L. var. sativa Plant Material
Source: Molecules. 2023 Dec 8;28(24):8008. doi: 10.3390/molecules28248008 (PMC10746033; doi:10.3390/molecules28248008)
Supplement: Supplementary file 1 [file molecules-28-08008-s001.zip › molecules-2742345-supplementary.pdf]

Table S1. Intra- and inter-day precision data of the determination of cannabinoids in inflorescences, leaves, and CRM (Intra-day n=6; Inter-day, day=3, n=3 days × 6=18)

	Nominal concentration [μg mL^{-1}]	Inflorescences			Leaves			CRM		
		Measured concentration [μg mL^{-1}]	[1]R (%)	[2]RSD (%)	Measured concentration [μg mL^{-1}]	R (%)	RSD (%)	Measured concentration [μg mL^{-1}]	R (%)	RSD (%)
					**CBD**					
**Intra-day**	**0.200**	0.200	100	1	0.200	100	1	0.200	100	1
	**3.200**	3.190	99	3	3.180	99	3	3.190	99	3
	**25.600**	25.490	99	5	25.550	99	2	25.450	99	2
**Inter-day**	**0.200**	0.190	95	4	0.190	95	6	0.210	105	6
	**3.200**	3.180	99	3	3.170	99	8	3.220	101	8
	**25.60**	25.190	98	2	25.280	98	6	25.620	100	6
					**CBN**					
**Intra-day**	**0.008**	0.008	100	1	0.008	100	3	0.008	100	4
	**0.128**	0.127	99	2	0.129	100	5	0.130	101	7
	**1.024**	1.022	99	3	1.024	100	3	1.024	100	3
**Inter-day**	**0.008**	0.008	100	4	0.008	100	5	0.008	100	5
	**0.128**	0.127	99	3	0.126	98	5	0.126	98	2
	**1.024**	1.022	99	2	1.022	99	3	1.024	100	2
					**CBG**					
**Intra-day**	**0.008**	0.008	100	1	0.008	100	4	0.008	100	1
	**0.128**	0.126	98	2	0.127	99	6	0.128	100	4
	**1.024**	1.019	99	2	1.022	99	1	1.026	100	2
**Inter-day**	**0.008**	0.008	100	1	0.008	100	3	0.008	100	2
	**0.128**	0.125	97	3	0.124	97	5	0.130	102	8
	**1.024**	1.015	99	1	1.010	98	9	1.300	100	5
					**Δ^9-THC**					
**Intra-day**	**0.080**	0.080	100	1	0.080	100	2	0.080	100	2
	**1.280**	1.280	100	2	1.270	100	4	1.290	100	5
	**10.240**	10.240	100	2	10.250	100	2	10.250	100	6
**Inter-day**	**0.080**	0.080	100	3	0.081	101	4	0.081	101	3
	**1.280**	1.230	96	4	1.200	94	1	1.260	98	4
	**10.240**	10.250	100	4	10.230	99	2	10.290	100	1
					**Δ^8-THC**					
**Intra-day**	**0.008**	0.008	100	3	0.008	100	4	0.008	100	3
	**0.128**	0.128	100	4	0.128	100	1	0.127	99	5
	**1.024**	1.022	99	3	1.022	99	8	1.020	99	5
**Inter-day**	**0.008**	0.008	100	2	0.008	100	3	0.007	88	5
	**0.128**	0.128	100	1	0.120	94	4	0.123	96	6
	**1.024**	1.020	99	3	1.019	99	5	1.018	99	9
					**CBC**					
**Intra-day**	**0.020**	0.020	100	1	0.022	110	7	0.018	90	4
	**0.320**	0.320	100	2	0.318	99	5	0.319	99	4
	**2.560**	2.560	100	3	2.550	99	2	2.550	99	9
**Inter-day**	**0.020**	0.020	100	1	0.019	95	4	0.017	85	8
	**0.320**	0.310	97	3	0.316	98	3	0.318	99	7
	**2.560**	2.540	99	1	2.500	97	2	2.540	99	1
					**CBL**					
**Intra-day**	**0.020**	0.020	100	1	0.020	100	5	0.021	100	6
	**0.320**	0.320	100	1	0.322	100	7	0.321	100	1
	**2.560**	2.560	100	2	2.540	99	4	2.520	98	4
**Inter-day**	**0.020**	0.020	100	2	0.019	95	5	0.018	90	10
	**0.320**	0.330	103	6	0.318	99	6	0.316	98	5
	**2.560**	2.540	99	3	2.520	98	5	2.500	98	8
					**CBDV**					
**Intra-day**	**0.008**	0.008	100	1	0.008	100	4	0.008	100	3
	**0.128**	0.125	97	4	0.124	97	4	0.122	95	4
	**1.024**	1.023	99	4	1.022	99	1	1.020	99	3
**Inter-day**	**0.008**	0.008	100	2	0.008	100	4	0.008	100	3
	**0.128**	0.124	97	4	0.122	95	8	0.120	94	7
	**1.024**	1.026	98	5	1.020	99	4	1.018	99	6
					**Δ^9-THCV**					

Intra-day	**0.008**	0.008	100	1	0.008	100	4	0.008	100	4
	**0.128**	0.127	99	2	0.124	97	8	0.122	95	7
	**1.024**	1.022	99	2	1.020	99	8	1.018	99	8
Inter-day	**0.008**	0.008	100	3	0.008	100	1	0.008	100	2
	**0.128**	0.127	99	2	0.120	94	6	0.118	92	8
	**1.024**	1.022	99	4	1.018	99	2	1.017	99	3
**CBDA**										
Intra-day	**0.400**	0.390	98	4	0.370	96	8	0.415	101	3
	**6.400**	6.380	99	2	6.380	101	1	6.420	101	2
	**51.200**	51.180	99	3	51.150	99	4	51.115	101	5
Inter-day	**0.400**	0.370	96	6	0.360	95	5	0.390	99	2
	**6.400**	6.370	99	4	6.340	99	3	6.390	99	3
	**51.200**	51.150	99	2	51.100	98	4	51.190	99	3
**CBNA**										
Intra-day	**0.002**	0.002	100	2	0.002	100	2	0.002	100	3
	**0.032**	0.032	100	2	0.30	94	6	0.30	94	8
	**0.256**	0.256	100	1	0.250	98	4	0.249	97	3
Inter-day	**0.002**	0.002	100	3	0.002	100	2	0.002	100	2
	**0.032**	0.031	97	7	0.029	96	4	0.028	93	7
	**0.256**	0.253	98	5	0.0248	96	8	0.245	95	6
**CBGA**										
Intra-day	**0.020**	0.020	100	2	0.020	100	2	0.022	100	3
	**0.320**	0.320	100	3	0.323	101	1	0.330	103	2
	**2.560**	2.560	100	3	2.450	96	4	2.560	100	2
Inter-day	**0.020**	0.020	100	4	0.019	95	7	0.018	90	10
	**0.320**	0.310	97	6	0.300	94	6	0.315	98	5
	**2.560**	2.540	99	4	2.400	94	5	2.500	98	7
**Δ^9-THCA-A**										
Intra-day	**0.080**	0.080	100	1	0.078	98	3	0.076	96	5
	**1.280**	1.260	98	2	1.250	97	3	1.270	99	4
	**10.240**	10.200	99	3	10.150	99	3	10.210	99	2
Inter-day	**0.080**	0.080	100	1	0.075	94	8	0.073	91	10
	**1.280**	1.260	98	2	1.230	96	6	1.235	96	5
	**10.240**	10.190	99	1	10.035	98	4	10.125	99	2
**CBCA**										
Intra-day	**0.080**	0.080	100	1	0.078	98	4	0.079	99	3
	**1.280**	1.270	99	2	1.260	98	4	1.290	101	5
	**10.240**	10.220	99	2	10.200	99	3	10.180	99	2
Inter-day	**0.080**	0.080	100	1	0.075	94	7	0.077	96	4
	**1.280**	1.270	99	3	1.240	97	5	1.250	98	5
	**10.240**	10.180	99	3	10.175	99	2	10.140	99	2
**CBLA**										
Intra-day	**0.020**	0.020	100	2	0.019	95	8	0.019	95	5
	**0.320**	0.320	100	2	0.318	99	3	0.316	98	3
	**2.560**	2.560	100	2	2.550	99	3	2.545	99	4
Inter-day	**0.020**	0.020	100	3	0.018	90	10	0.017	85	5
	**0.320**	0.320	100	4	0.316	98	4	0.315	98	6
	**2.560**	2.550	99	5	2.500	97	5	2.458	96	4
**CBDVA**										
Intra-day	**0.020**	0.020	100	2	0.020	100	2	0.020	100	2
	**0.320**	0.320	100	1	0.316	98	4	0.320	100	4
	**2.560**	2.560	100	3	2.560	100	2	2.560	100	1
Inter-day	**0.020**	0.020	100	1	0.018	90	10	0.018	90	8
	**0.320**	0.310	97	5	0.310	96	8	0.311	97	5
	**2.560**	2.540	99	6	2.457	96	4	2.320	91	4
**Δ^9-THCVA**										
Intra-day	**0.020**	0.020	100	1	0.018	90	7	0.018	90	8
	**0.320**	0.320	100	2	0.316	98	4	0.318	99	4
	**2.560**	2.560	100	1	2.456	96	2	2.564	101	2
Inter-day	**0.020**	0.020	100	2	0.016	80	8	0.016	80	8
	**0.320**	0.320	100	1	0.312	97	5	0.299	93	7
	**2.560**	2.550	99	4	2.412	94	4	2.218	87	6

[1]Method and [2]precision recovery values (expressed as % RSD) obtained by enriching each matrix (inflorescences, leaves, CRM) at three different levels for each compound. Recovery was determined based on the equation: % R = $[(C_f\text{-}C_u)*100]/C_a$, where C_a is the calculated (not analyzed) concentration of analyte added to the test sample; C_f is the concentration of fortified samples; C_u is the concentration for unfortified samples.

Table S2. Results obtained, average values and z-score for dried hemp proficiency tests (HFL2301)

Compound	Results (mg kg^{-1})	Average value (mg kg^{-1})	z-score
**CBD**	26,970	33,216	-1.5
**CBDA**	33,010	42,680	-1.4
**CBG**	4,520	4,930	-0.6
**CBGA**	27,520	43,280	-2.1
**Δ^9-THC**	2,070	2,120	-0.2
**Δ^9-THCA-A**	550	640	-1.0
**Δ^8-THC**	1.6	4	-0.5
**Δ^9-THCV**	40	70	-0.3
**Δ^9-THCVA**	370	-	-
**CBDV**	730	970	-0.5
**CBDVA**	2,060	2,170	-0.4
**CBN**	200	200	-0.1
**CBNA**	40	-	-
**CBC**	3,610	3,254	0.8
**CBCA**	680	3,060	-1.5
**CBL**	120	-	-
**CBLA**	116	-	-

Table S3. Results obtained, average values and z-score for dried hemp proficiency tests (HFL2305)

Compound	Results (mg kg^{-1})	Average value (mg kg^{-1})	z-score
**CBD**	1,981	1,861	0.5
**CBDA**	4,080	3,480	1.2
**CBG**	9,350	10,400	-0.8
**CBGA**	50,800	77,380	-
**Δ^9-THC**	720	630	0.8
**Δ^9-THCA-A**	270	220	0.4
**Δ^8-THC**	0	0	-
**Δ^9-THCV**	60	90	-0.3
**Δ^9-THCVA**	80	-	-
**CBDV**	310	280	0.2
**CBDVA**	1100	1260	-1.0
**CBN**	130	110	0.4
**CBNA**	1850	-	-

**CBC**	2240	2381	-0.6
**CBCA**	1180	1370	-1.0
**CBL**	20	-	-
**CBLA**	21	-	-

Table S4. The content of 17 cannabinoids for each hemp tea sample [mg kg $^{-1}$].

Sample	CBC	CBDV	CBG	CBL	CBN	CBNA	Δ^8-THC	Δ^9-THC	Δ^9-THCV	CBDVA	CBD	CBGA	Δ^9-THCVA	CBLA	CBCA	Δ^9-THCA-A	CBDA
**1**	262.5^{l}±16.9	35.4^{ijk}±6.4	155.3^{efghi}±17.8	38.0^{n}±7.9	80.0^{n}±10.3	19.7^{hij}±2.0	4.9^{bcdef}±0.8	122.6^{cdef}±14.0	0.9^{abc}±0.1	95.1^{bcd}±11.8	$5,299^{lmn}$±211	240.4^{ab}±16.3	8.8^{bcde}±1.3	40.2^{bcde}±3.2	115.0^{ghijk}±17.5	284.7^{bcde}±8.1	$8,877^{ghijkl}$±660
**2**	196.1^{ghi}±7.9	28.6^{fghi}±2.8	234.8^{ij}±21.3	14.8^{ijk}±1.6	30.5^{hij}±2.8	17.3^{fghi}±2.3	7.6^{fg}±0.2	223.7^{jk}±14.1	$1.9^{abcdefg}$±0.2	80.0^{bcdef}±3.5	$4,880^{klmn}$±749	716.5^{abcde}±110.6	7.9^{abcd}±1.1	65.5^{efgh}±10.6	122.2^{hijk}±4.6	331.6^{cdefgh}±38.10	$6,810^{defghijk}$±1,127
**3**	203.3^{ijk}±15.8	14.0^{bc}±1.5	2325^{m}±199	$10.6^{cdefghij}$±1.9	13.3^{bcd}±0.9	2.0^{a}±0.4	$<\mathrm{LOD}^{a}$	49.3^{ab}±4.2	0.5^{a}±0.1	12.7^{a}±1.2	$1,535^{abcde}$±205	$3,646^{i}$±671	4.5^{a}±1.4	10.0^{a}±1.1	32.5^{ab}±4.4	58.30^{a}±5.7	$1,904^{ab}$±292
**4**	240.0^{jkl}±15.1	26.3^{efgh}±2.9	416.5^{k}±32.5	$10.3^{bcdefghij}$±0.2	10.0^{abc}±0.2	8.0^{bc}±0.5	3.9^{bcd}±0.1	188.7^{hij}±15.0	2.4^{defgh}±0.1	100.0^{defgh}±4.0	$2,391^{bcdefgh}$±345	$1,797^{g}$±283	13.3^{ghij}±1.9	70.7^{fghi}±5.0	94.7^{efghi}±5.2	$374.8^{cdefghi}$±20.3	$7,534^{defghij}$±1,269
**5**	241.3^{jkl}±12.5	57.6^{l}±4.4	96.7^{bcdef}±6.7	11.8^{efghij}±1.0	87.3^{o}±7.9	22.0^{jklm}±1.6	$<\mathrm{LOD}^{a}$	616.8^{o}±31.2	23.8^{n}±1.1	144.7^{ij}±17.9	$4,428^{ijkl}$±279	218.7^{ab}±19.3	26.0^{l}±2.3	7.7^{a}±0.2	49.1^{abcd}±2.5	419.7^{ghij}±30.2	$9,070^{hijkl}$±1,262
**6**	126.0^{bcde}±14.1	20.7^{cdef}±1.7	210.9^{hi}±25.7	$10.5^{bcdefghij}$±0.2	20.7^{efg}±1.2	12.0^{cdefg}±1.5	5.1^{bcdef}±0.6	160.8^{ghi}±9.5	$2.0^{bcdefgh}$±0.4	62.7^{bcd}±5.2	$2,663^{cdefgh}$±312	$1,155^{defg}$±172	6.7^{abcd}±1.4	48.0^{cdef}±3.0	84.0^{defg}±5.1	292.9^{bcdef}±46.9	$9,436^{ijkl}$±2,724
**7**	190.7^{efgh}±25.1	24.1^{defgh}±1.8	203.6^{ghi}±16.5	$10.2^{bcdefghij}$±1.5	31.3^{hijk}±3.7	28.7^{mno}±3.1	5.0^{bcdef}±1.9	183.3^{ghi}±15.5	1.6^{abcdef}±0.2	88.0^{cdef}±1.8	$4,516^{jkl}$±109	890.0^{bcdef}±69.2	$12.7^{defghij}$± 1.5	124.0^{k}±10.6	129.9^{jk}±11.3	616.7^{m}±34.9	$10,457^{kl}$±1,071
**8**	145.3^{defg}±14.9	26.1^{efgh}±2.6	164.3^{fghi}±32.8	12.5^{fghij}±1.0	28.0^{hij}±3.4	22.7^{ijkl}±1.8	$<\mathrm{LOD}^{a}$	229.4^{jkl}±11.6	$2.0^{bcdefgh}$±0.2	83.3^{cdef}±7.7	$3,622^{fghijkl}$±144	386.7^{abc}±14.8	10.0^{cdef}±0.7	90.7^{ghij}±7.8	133.5^{jk}±20.4	480.0^{ijkl}±29.1	$8,024^{defghijk}$±946
**9**	216.7^{ijk}±5.7	30.1^{ghij}±4.0	299.1^{j}±32.8	15.1^{jk}±2.1	35.3^{jk}±2.2	27.3^{klmn}±1.4	$<\mathrm{LOD}^{a}$	250.0^{kl}±9.8	2.7^{defgh}±0.1	110.7^{efghi}±12.1	$5,004^{klmn}$±211	$1,592^{fg}$±158	10.7^{defgh}±1.0	104.0^{hij}±13.1	204.0^{l}±20.0	532.0^{jklm}±36.1	$9,746^{jkl}$±1,195
**10**	112.7^{bc}±9.5	15.0^{bc}±2.0	132.9^{defgh}±15.6	$11.7^{defghij}$±0.3	16.7^{cde}±1.6	21.5^{ijk}±3.0	$<\mathrm{LOD}^{a}$	148.7^{defg}±10.0	$1.7^{abcdefg}$±0.1	91.3^{cdefg}±8.3	$2,225^{bcdefgh}$±133	$1,292^{efg}$±358	14.7^{fghij}±1.7	105.3^{hij}±15.3	144.7^{k}±5.8	534.0^{jklm}±33.4	$5,809^{defgh}$±1,113
**11**	257.3^{jkl}±31.6	20.2^{cdef}±3.9	109.6^{cdef}±9.8	$11.5^{defghij}$±2.0	28.2^{hijk}±0.6	19.3^{ghij}±1.5	6.6^{defg}±1.8	171.3^{fgh}±20.6	1.5^{abcde}±0.2	68.0^{bcdef}±12.3	$3,873^{ghijkl}$±415	224.3^{ab}±86.1	10.7^{cdefg}±1.3	110.7^{ijk}±19.0	148.0 ^{k}±11.5	617.3^{m}±41.6	$8,188^{efghijkl}$±1,466
**12**	155.3^{efg}±20.0	15.9^{bcd}±3.4	121.5^{cdefg}±17.4	$11.6^{defghij}$±1.0	26.0^{fghi}±2.8	10.7^{cde}±1.0	5.0^{bcdef}±0.6	92.7^{cde}±16.0	0.8^{abc}±0.2	43.3^{ab}±8.4	$2,686^{cdefghi}$±314	108.4^{a}±14.8	5.3^{ab}±0.9	61.3^{cdefg}±8.0	90.7^{efghi}±5.5	404.0^{fghi}±38.2	$5,036^{bced}$±254
**13**	150.7^{cdef}±10.5	18.3^{cde}±0.2	111.7^{cdef}±61.5	6.8^{abcdef}±1.4	23.2^{efgh}±3.0	9.9^{cd}±1.2	2.6^{b}±0.7	87.3^{bc}±10.2	0.7^{ab}±0.1	47.3^{abc}±8.4	$3,016^{efghij}$±152	133.2^{a}±14.4	6.0^{ab}±0.4	54.7^{cdef}±10.2	86.2^{efgh}±16.6	398.0^{efghi}±20.0	$4,793^{bcde}$±815
**14**	289.7^{l}±5.8	13.2^{bc}±1.0	404.5^{k}±39.9	11.9^{efghij}±0.6	13.3^{bcd}±2.9	14.8^{efghi}±1.9	3.4^{bc}±0.5	119.2^{cdef}±6.4	0.9^{abc}±0.1	80.0^{bcdef}±8.0	$2,669^{cdefghi}$±512.8	$3,060^{hi}$±187	20.0^{k}±2.6	107.9^{jk}±15.9	216.4^{l}±4.4	948.7^{n}±83.6	$8,046^{defghijk}$±1,015
**15**	160.5^{def}±13.8	15.7^{bcd}±1.1	135.1^{defgh}±3.7	$8.3^{abcdefgh}$±0.9	21.7^{efg}±1.5	11.3^{cdef}±0.8	5.0^{bcdef}±0.2	106.8^{cd}±6.4	0.6^{a}±0.2	50.0^{abc}±8.5	$2,710^{defgh}$±62.6	265.2^{ab}±40.1	5.8^{abc}±0.4	59.1^{defg}±4.4	92.1^{ghij}±1.9	440.1^{hijk}±22.3	$5,329^{cedfg}$±809
**16**	211.8^{hij}±7.5	8.1^{a}±0.6	404.8^{k}±15.2	5.9^{abcd}±0.2	7.3^{ab}±0.3	10.0^{cde}±1.4	3.2^{b}±0.7	84.9^{bc}±6.7	0.8^{abc}±0.2	58.7^{bcd}±8.0	$1,835^{abcdef}$±238.8	$2,679^{h}$±692	14.8^{jk}±2.5	94.9^{ij}±4.6	110.3^{ghijk}±9.8	634.7^{m}±28.7	$6,090^{defghi}$±476
**17**	102.3^{b}±2.7	4.0^{a}±0.2	$2,032^{l}$±93	4.7^{ab}±0.1	4.3^{a}±0.1	3.9^{ab}±0.2	$<\mathrm{LOD}^{a}$	21.4^{a}±1.2	0.5^{a}±0.1	14.7^{a}±1.2	308.0^{a}±14.8	$2,730^{h}$±247.6	6.5^{ab}±0.1	12.7^{ab}±2.6	16.7^{a}±2.1	196.0^{b}±10.6	501.2^{a}±83.8
**18**	54.8^{a}±4.0	21.3^{cdefg}±1.5	21.6^{ab}±3.2	$9.1^{abcdefghi}$±0.3	46.6^{l}±2.1	32.4^{no}±0.7	4.3^{bcde}±0.6	148.9^{efg}±4.2	5.2^{k}±0.5	89.3^{cdef}±14.5	$1,016.7^{abcd}$±88.2	104.1^{a}±36.1	17.1^{hij}±0.2	31.3^{abcd}±8.2	47.3^{abcd}±3.7	285.3^{bcde}±34.8	$2,173^{abc}$±326.2
**19**	255.6^{kl}±9.9	32.6^{hij}±0.2	76.5^{abcde}±2.7	23.7^{lm}±0.5	39.5^{kl}±0.6	21.9^{jklmn}±0.8	6.1^{cdefg}±1.0	178.8^{ghi}±10.2	19.3^{m}±1.2	92.0^{cdefg}±14.0	$4,586^{jklm}$ ±590	107.0^{a}±12.6	8.3^{bcde}±0.5	58.7^{defg}±3.5	149.2^{k}±6.1	$371.3^{cdefghi}$±22.0	$6,671^{defghij}$±724
**20**	38.2^{a}±1.7	14.6^{bc}±1.3	8.1^{a}±0.6	3.4^{a}±0.2	33.5^{ijk}±1.4	27.8^{lmn}±1.4	3.2^{b}±0.2	93.5^{bc}±3.1	3.6^{hij}±0.2	71.3^{bcde}±2.3	707.8^{ab} ±60.9	26.9^{a}±4.2	13.1^{efghij}±0.1	26.6^{abc}±5.4	33.2^{abc}±2.8	262.7^{bc}±28.9	$1,594^{ab}$±244
**21**	46.3^{a}±1.7	17.6^{cde}±1.4	9.7^{a}±0.9	4.9^{abc}±0.5	46.5^{l}±0.5	39.3^{p}±2.7	2.5^{b}±0.3	119.4^{cde}±3.1	4.3^{jk}±0.1	93.3^{cdfeg}±11.0	911.5^{abc} ±81.0	33.3^{a}±8.4	17.9^{jk}±1.3	33.1^{abcd}±8.5	46.1^{abcd}±1.9	292.7^{bcdef}±28.5	$2,055^{abc}$±200
**22**	314.0^{m}±28.1	37.4^{ijk}±1.9	131.3^{defgh}±3.1	19.5^{kl}±0.2	55.5^{m}±3.3	28.9^{op}±0.9	8.5^{g}±2.4	224.5^{jk}±10.3	2.1^{cdefg}±0.2	139.3^{hij}±14.3	$6,359^{mn}$ ±276	246.4^{ab}±36.9	12.1^{efghij}±1.5	86.7^{ijk}±6.5	188.5^{l}±10.6	572.0^{lm}±10.6	$6,736^{defghij}$±708
**23**	169.9^{efgh}±4.4	21.3^{cdefg}±1.2	43.8^{abc}±0.7	12.7^{ghij}±1.5	19.3^{def}±0.9	10.1^{cde}±0.6	$<\mathrm{LOD}^{a}$	140.7^{defg}±7.9	1.1^{abcd}±0.1	84.0^{bcdef}±13.0	$3,968^{hijkl}$ ±421	37.7^{a}±6.2	3.5^{a}±0.1	32.1^{abcd}±0.8	69.3^{cdef}±5.5	283.3^{bcd}±28.7	4.509^{bcd}±591
**24**	283.7^{lm}±3.3	41.6^{k}±2.4	62.1^{abcd}±0.6	26.8^{m}±1.1	24.6^{efgh}±1.2	15.1^{defgh}±1.9	6.9^{efg}±0.8	278.2^{lm}±3.9	2.6^{efgh}±0.2	106.0^{cdefg}±13.3	$6,542^{n}$ ±185	39.3^{a}±4.0	5.0^{ab}±0.8	41.6^{cde}±0.2	136.5^{jk}±6.1	312.7^{cdefg}±9.9	5.886^{defghi}±789
**25**	117.7^{bcd}±10.1	21.0^{cdef}±1.2	126.2^{cdefg}±6.7	$7.9^{abcdefg}$±0.6	6.9^{ab}±0.2	11.9^{cdefg}±0.8	$<\mathrm{LOD}^{a}$	139.8^{defg}±5.1	1.1^{abcd}±0.1	133.3^{ghij}±22.9	$2,164^{bcdefg}$ ±93	$1,088^{cdefg}$±146	14.3^{ij}±0.3	74.1^{fghi}±1.4	141.9^{jk}±15.4	553.3^{klm}±52.6	$8.500^{fghijkl}$±178

**26**	134.4^{bcde}±3.1	31.3^{hij}±1.8	124.3^{cdefg}±7.9	6.7^{abcd}±1.5	10.9^{abc}±0.3	13.5^{cdefg}±0.5	3.6^{bc}±0.2	177.1^{ghi}±0.3	$1.7^{abcdefg}$±0.1	164.0^{j}±10.6	$2,860^{efghij}$ ± 502	540.7^{abcde}±33.1	14.0^{fghij}±0.7	60.9^{defg}±3.9	138.9^{jk}±9.6	430.7^{hij}±22.5	$7.370^{defghijk}$±1,063
**27**	157.5^{defg}±5.6	15.9^{bcd}±0.8	137.6^{defgh}±1.7	$8.4^{abcdefgh}$±1.3	11.6^{abcd}±0.6	15.3^{defgh}±1.3	4.2^{bcde}±0.5	159.6^{fgh}±8.3	2.9^{fghi}±0.3	113.3^{fghi}±5.9	$2,219^{bcdefgh}$ ± 417	486.0^{abcd}±38.2	14.8^{ghij}±1.6	92.6^{hij}±7.5	125.2^{ijk}±4.5	572.7^{lm}±44.3	$9,071^{hijkl}$±642
**28**	186.5^{fghi}±14.7	38.7^{jk}±3.8	76.9^{abcde}±5.2	$8.0^{abcdefgh}$±1.4	11.6^{abcd}±0.4	10.7^{cde}±1.6	<LOD^{a}	206.3^{ij}±9.7	4.1^{ijk}±0.1	216.3^{k}±17.4	$3,494^{fghijk}$ ± 361	228.7^{ab}±47.3	25.1^{l}±4.3	81.5^{ghij}±7.2	105.6^{fghij}±7.4	545.3^{klm}±26.4	$8,936^{hijkl}$±627
**29**	44.3^{a}±7.0	87.5^{m}±5.1	58.9^{abcd}±1.8	$7.4^{abcdefg}$±1.3	32.9^{ijk}±0.8	17.1^{fghi}±0.6	<LOD^{a}	347.3^{n}±27.8	13.3^{l}±0.7	280.0^{l}±29.3	$2,812^{defghij}$ ± 224	132.0^{a}±4.0	39.4^{m}±0.7	50.0^{cdef}±3.6	65.0^{bcde}±2.9	384.7^{defghi}±13.6	$5,301^{cdef}$±1,152
**30**	111.7^{bc}±14.8	42.3^{k}±3.9	167.1^{fghi}±2.4	13.7^{hij}±1.2	33.9^{ijk}±1.6	22.1^{ijkl}±0.6	4.7^{bcde}±0.5	295.6^{m}±10.5	3.0^{ghij}±0.2	117.0^{fghi}±14.7	$6,448^{n}$ ± 1,825	189.3^{ab}±24.2	11.4^{defghi}±0.4	73.8^{fghi}±4.7	125.9^{ijk}±22.2	470.0^{ijkl}±15.1	$11,692^{l}$±1,443

$^{a-o}$ *– values within columns followed by the same letter are not significantly different ac-cording for α = 0.01*
